# SHP-1 knockdown suppresses mitochondrial biogenesis and aggravates mitochondria-dependent apoptosis induced by all trans retinal through the STING/AMPK pathways

**DOI:** 10.1186/s10020-022-00554-w

**Published:** 2022-10-22

**Authors:** Xiaonan Zhuang, Jun Ma, Gezhi Xu, Zhongcui Sun

**Affiliations:** 1 Department of Ophthalmology, Eye and ENT Hospital of Fudan University, 83 Fenyang Road, Shanghai, 200031 China; 2 Eye Institute, Eye & ENT Hospital, Fudan University, Shanghai, China; 3 Shanghai Key Laboratory of Visual Impairment and Restoration, Fudan University, Shanghai, China; 4 NHC Key Laboratory of Myopia, Fudan University, Shanghai, China

**Keywords:** Age-related macular degeneration, Retinal pigment epithelium, All trans retinal, Src-homology 2 domain-containing phosphatase-1, Stimulator of interferon genes, Adenosine monophosphate-activated protein kinase, Mitochondrial biogenesis

## Abstract

**Background:**

Oxidative stress-caused damage to the retinal pigment epithelium (RPE) underlies the onset and progression of age-related macular degeneration (AMD). Impaired mitochondrial biogenesis sensitizes RPE cells to mitochondrial dysfunction, energy insufficiency and death. Src-homology 2 domain-containing phosphatase (SHP)-1 is important in regulating immune responses and cell survival. However, its roles in cell survival are not always consistent. Until now, the effects of SHP-1 on RPE dysfunction, especially mitochondrial homeostasis, remain to be elucidated. We sought to clarify the effects of SHP-1 in RPE cells in response to atRAL-induced oxidative stress and determine the regulatory mechanisms involved.

**Methods:**

In the all trans retinal (atRAL)-induced oxidative stress model, we used the vector of lentivirus to knockdown the expression of SHP-1 in ARPE-19 cells. CCK-8 assay, Annexin V/PI staining and JC-1 staining were utilized to determine the cell viability, cell apoptosis and mitochondrial membrane potential. We also used immunoprecipitation to examine the ubiquitination modification of stimulator of interferon genes (STING) and its interaction with SHP-1. The expression levels of mitochondrial marker, proteins related to mitochondrial biogenesis, and signaling molecules involved were examined by western blotting analysis.

**Results:**

We found that SHP-1 knockdown predisposed RPE cells to apoptosis, aggravated mitochondrial damage, and repressed mitochondrial biogenesis after treatment with atRAL. Immunofluoresent staining and immunoprecipitation analysis confirmed that SHP-1 interacted with the endoplasmic reticulum-resident STING and suppressed K63-linked ubiquitination and activation of STING. Inhibition of STING with the specific antagonist H151 attenuated the effects of SHP-1 knockdown on mitochondrial biogenesis and oxidative damage. The adenosine monophosphate-activated protein kinase (AMPK) pathway acted as the crucial downstream target of STING and was involved in the regulatory processes.

**Conclusions:**

These findings suggest that SHP-1 knockdown potentiates STING overactivation and represses mitochondrial biogenesis and cell survival, at least in part by blocking the AMPK pathway in RPE cells. Therefore, restoring mitochondrial health by regulating SHP-1 in RPE cells may be a potential therapeutic strategy for degenerative retinal diseases including AMD.

## Introduction

Degenerative retinal diseases, including age-related macular degeneration (AMD) and retinitis pigmentosa, are a group of eye diseases that cause incurable blindness (Wong et al. 2014; Verbakel et al. 2018). The visual cycle takes place between the retinal pigment epithelial (RPE) cells and photoreceptors, and it is necessary for normal vision in the vertebrate retina (Kiser et al. 2012). All trans retinal (atRAL) is a byproduct of the visual cycle that is generated from visual chromophore 11-cis-retinal following light exposure. atRAL carries a highly reactive aldehyde group and causes retinal toxicity unless it is eliminated in time. In a transgenic mouse model with defective atRAL clearance, the aberrant accumulation of atRAL leads to rapidly progressive retinal degeneration (Maeda et al. 2008, 2009). The degenerated RPE in this model shows excessive deposition of lipofuscin and drusen, a typical characteristic of the retina in humans with AMD. Generally, oxidative stress-induced dysfunction and the loss of RPE cells are pivotal to the pathogenesis of AMD. The mitochondria in RPE cells are particularly vulnerable to various pathological stimuli, including light and atRAL (Nunez-Alvarez et al. 2019; Li et al. 2015). Mitochondrial damage leads to an energy crisis, mitochondria-dependent apoptosis and eventually the loss of RPE cells (Brown et al. 2019; Nashine et al. 2017). In addition, mitochondrial dysfunction is exacerbated by dysregulation of mitochondrial quality control, including mitochondrial biogenesis, dynamics (fission/fusion) and mitophagy (Kaarniranta et al. 2020). These pathological events provide the rationale for the protection and restoration of mitochondrial homeostasis in RPE cells, especially before retinal photoreceptors undergo irreversible damage in degenerative retinal diseases. Therefore, the regulatory mechanisms of mitochondrial homeostasis in the RPE warrant further investigation.

Src-homology 2 domain-containing phosphatase (SHP)-1, encoded by the gene *protein-tyrosine phosphatase non-receptor 6 (PTPN6)*, has been widely studied owing to its roles in cellular adhesion, chemotaxis, inflammatory responses, and cell survival (Chong and Maiese 2007). Although SHP-1 plays a proapoptotic role in neutrophils (Yousefi and Simon 2003; Jia et al. 2008), and in a myocardial infarction model (Kim et al. 2013), it was demonstrated to inhibit apoptosis in a renal ischemia–reperfusion injury model (Tian et al. 2019). It was reported that SHP-1 deficiency leads to the onset of retinal degeneration in mice by 3 weeks age, followed by a rapid progressive loss of photoreceptors (Lyons et al. 2006). SHP-1 was also demonstrated to be present in D407 cells, a human RPE cell line (Papadaki et al. 2010). However, the mechanisms underlying the effects of SHP-1 in the retina, especially in RPE cells, remain to be elucidated.

Growing evidence supports the implications of stimulator of interferon genes (STING) activation in cell death, including mitochondria-dependent apoptosis (Petrasek et al. 2013; Zhang et al. 2021). STING, also known as MITA or TMEM173, is an adaptor protein residing in endoplasmic reticulum (ER), and actively participates in eliciting innate immune responses to cystolic DNA (Liu et al. 2015). Aberrant STING activation was also observed in the degenerating RPE in animal models and human eyes affected by AMD (Kerur et al. 2018). The aggregation and activation of STING are regulated by a series of posttranslational modification including phosphorylation (Wang et al. 2020) and ubiquitination (Liu et al. 2017). Previous studies have shown that SHP-1 inhibits the phosphorylation and subsequent K63-linked ubiquitination for protein activation (Zhou et al. 2019; Hao et al. 2020). These findings raise the possibility that the pathological activation of STING may be regulated by SHP-1 in the diseased RPE cells.

The present study aims to investigate the effects of SHP-1 knockdown on mitochondrial homeostasis and survival of RPE cells following stimulation with atRAL and elucidate the underlying mechanisms. We reveal that SHP-1 knockdown represses mitochondrial biogenesis and aggravates mitochondria-dependent apoptosis in RPE cells exposed to atRAL, a process that is at least partly mediated by the STING-adenosine monophosphate-activated protein kinase (AMPK) pathways. The depressed mitochondrial metabolic capacity after SHP-1 knockdown increases the susceptibility of RPE cells to oxidative stress-induced death. These findings may provide a novel approach to maintain mitochondrial homeostasis and aid the survival of RPE cells.

## Materials and methods

### Cell culture, DNA construction, transfection and drug administration

The immortal human retinal pigment epithelium cell line ARPE-19 was purchased from ATCC (Manassas, VA, USA). The cells were cultured in Dulbecco’s modified Eagle’s medium /Nutrient Mixture F-12 (DMEM/F-12) supplemented with 10% fetal bovine serum in a humidified incubator at 37 °C under 5% CO_2_. To knockdown SHP-1 in ARPE-19 cells, we used short hairpin RNA specific to human SHP-1 recombinant lentivirus (shRNA-SHP-1-rLV; Taitool Bioscience, Shanghai, China). The target sequence of human SHP-1 was 5’- CCTTGAGCAGGGTCTCTGCATCC-3’. The scramble sequence was 5’- CGCTGAGTACTTCGAAATGTC-3’. The sequences were inserted into the pLentai-hU6-shRNA (or scramble)-hEF1α-PuroR lentiviral vector. ARPE-19 cells were incubated with medium containing the virus (multiplicity of infection = 15) for 24 h. Then, the medium was refreshed, and the cells were cultured for another 48 h. The cell lysates were collected to determine the SHP-1 expression levels by western blotting. ARPE-19 cells transfected with scramble-rLV were used as control. atRAL(Sigma, St.Louis, MO, USA), a STING covalent antagonist (H151; Selleck Chemicals, Houston, TX, USA) and an AMPK inhibitor (Compound C; Selleck Chemicals), were dissolved and stored in the dark at -80 °C until use. The reagents were diluted with culture medium to an appropriate concentration immediately before use.

### Cell viability assay

The same numbers of cells were first seeded in 96-cell microplates. When cell confluence had reached 80%, the pretreated or untreated cells were incubated with culture medium supplemented with different concentrations of atRAL for 24 h. The medium was then replaced with fresh medium mixed with Cell Counting Kit-8 (CCK-8) reagents (Dojindo, Kumamoto, Japan). After incubation for 2 h, the color in each well was detected and analyzed using multimode microplate reader (Spark, Tecan, Männedorf, Zürich, Switzerland).

### Cell ATP assay

A CellTiter Glo Assay (Promega, Madison, WI, USA) was used to measure the ATP level in ARPE-19 cells. After equilibration at room temperature for 30 min, 100 μL of CellTiter Glo reagent was added to each well of white 96-well microplates. The microplates were immediately shaken for 2 min for complete cell lysis and the reaction. The microplates were then incubated for another 10 min at room temperature to stabilize the luminescence, which was measured using the microplate reader (Tecan).

### Annexin V-fluorescein isothiocyanate (FITC)/Propidium Iodide (PI) staining

An Annexin V-FITC Apoptosis Detection Kit (BD Biosciences, San Diego, CA, USA) was used to measure apoptosis. Briefly, after being washed twice with cold phosphate-buffered saline (PBS), the cells were digested with accutase (Invitrogen, Carlsbad, CA, USA) at room temperature and centrifuged at 300 g for 5 min for harvest. Then the cells were resuspended in binding buffer containing Annexin V-FITC and PI. The cell suspensions were gently vortexed and incubated in the dark for 15 min. Finally, flow cytometry was performed using a MoFlo XDP flow cytometer (Beckman Coulter, Miami, FL, USA) and analyzed using FlowJo software (TreeStar, Ashland, OR, USA).

### Mitochondrial Membrane Potential (MMP) Assay

A decrease in MMP is regarded as an early event in mitochondria-dependent apoptosis. We measured MMP using the JC-1 staining kit (Beyotime, Shanghai, China) according to the manufacturer’s instruction. In this assay, if MMP decreases, the fluorescence of JC-1 shifts from red (aggregated) to green (monomeric). Briefly, after being treated with atRAL for 12 h, the cells were refreshed with culture medium and an equal amount of JC-1 staining working solution at 37 °C for 20 min. The cells were then washed twice with ice-cold JC-1 staining buffer solution. Finally, the cells were placed in complete culture medium and viewed under an inverted fluorescence microscope (Leica, Wetzlar, Germany).The mean fluorescence intensities (red and green) were measured using ImageJ software (National Institutes of Health, Bethesda, MD, USA).

### Immunocytochemistry, mitochondria labeling, and mitochondria morphology analysis

The ARPE-19 cells were seeded onto coverslips, cultured, and treated with the specific drugs. The coverslips were then fixed with 4% formaldehyde at room temperature for 15 min. After being rinsed in PBS briefly, the coverslips were blocked and permeabilized in 5% goat serum and 0.3% Triton X-100 for 30 min. Next, the coverslips were incubated with the following primary antibodies at 4 °C overnight: mouse anti-SHP-1(610125, BD Biosciences); rabbit anti-calnexin (2679, Cell Signaling Technology, Danvers, MA, USA); rabbit anti-STING (198151-1-AP, Proteintech, Chicago, IL, USA); and rabbit anti-translocase of outer mitochondrial membrane 20 (TOMM20) (ab186735, abcam, Cambridge, UK). The coverslips were then incubated with Alexa Fluor 488-goat anti-mouse or Alexa Fluor 555-goat anti-rabbit secondary antibodies (Invitrogen) for 1 h at room temperature. After brief rinses, nuclei of cells were stained with 4',6-diamidino-2-phenylindole (DAPI) mounting medium (ab104139, Abcam). Finally, immunofluorescent images were taken with a laser confocal microscope (Leica).

To assess mitochondrial morphology, the acquired images were quantitatively analyzed with Mitochondria Analyzer, a publicly available plugin for ImageJ software as previously described (Chaudhry et al. 2020). The parameters included aspect ratio (AR) and form factor (FF) values. An AR value (the ratio of the length of major to minor axis) of 1 represents a circle. The AR value increases with elongation of the mitochondrion. An FF value (perimeter^2^/4π × area) of 1 indicates a simple unbranched mitochondrion. The FF value increases as the mitochondrial network develops into a more complex structure with more branching/elongation.

The abundance of mitochondria in live ARPE-19 cells was also estimated by MitoTracker Deepred (Dojindo) labeling. Briefly, the ARPE-19 cells were incubated with culture medium containing 1 μM of MitoTracker Deepred at 37 °C for 30 min. After being rinsed with PBS, the cells were digested with accutase and the cell suspensions were immediately analyzed by flow cytometry. The mean MitoTracker fluorescence intensities were measured using FlowJo software (TreeStar).

### Mitochondrial DNA (mtDNA) copy number

Genomic DNA, including mtDNA, was extracted from ARPE-19 cells using TIANamp Genomic DNA Kit (Tiangen Biotech, Beijing, China), and the mtDNA copy number was evaluated by real-time quantitative PCR. Real-time quantitative PCR was performed using the CFX RT-qPCR detection system (Bio-Rad, Hercules, CA, USA) with the following reaction conditions: 95 °C for 10 min (pre-degeneration), and 40 cycles of 95 °C for 15 s (degeneration) and 60 °C for 30 s (annealing and extension). The melting curves were obtained. The mitochondria-encoded NADH dehydrogenase subunit 1 (*mt-ND1*), and mitochondria-encoded cytochrome c oxidase III (*mt-CO3*) were chosen to represent mtDNA, and *β-actin* was chosen to represent nuclear DNA (nDNA). The following primers sequences were used (forward and reverse): *mt-ND1*, ATACCCATGGCCAACCTCCT and GGGCCTTTGCGTAGTTGTAT; *mt-CO3*, ATGACCCACCAATCACATGC and ATCACATGGCTAGGCCGGAG; *β-actin*, CGAGAAGATGACCCAGGTGAGT and GAGAGACAAACACCAGAAAAAGAGC. The mtDNA copy number was determined by analyzing the ΔΔCt for *mt-ND1* and *mt-CO3* normalized to *β-actin*.

### Western blotting and immunoprecipitation

The ARPE-19 cells were lysed in lysis buffer (Beyotime) supplemented with phenylmethylsulfonyl fluoride and protease inhibitor cocktail (Beyotime) on ice. The lysates, after brief ultrasonic treatment, were centrifuged at 13.6 × 10^3^ g for 5 min at 4 ℃. The protein concentration of the collected supernatants was determined using a BCA protein assay kit (Beyotime). The supernatants were mixed with the 5 × sample loading buffer and boiled at 95 °C for 5 min.

For immunoprecipitation, the supernatants were first incubated with the antibody on a shaker at 4 °C overnight. Then AG beads (Millipore, Billerica, MA, USA) were added to the supernatant and incubated at 4 ℃ for 2 h. The mixture was centrifuged at 4 °C and rinsed gently with PBS-Tween buffer three times. The precipitates were boiled with loading buffer as mentioned above. The samples were then separated by sodium dodecyl sulphate–polyacrylamide gel electrophoresis and transferred to a polyvinylidene difluoride membrane (Millipore). After being blocked with 5% nonfat milk for 1 h, the membranes were incubated with the following primary antibodies at 4 °C overnight: rabbit anti-SHP-1(ab32559, abcam); rabbit anti-TOMM20 (ab186725, abcam); rabbit anti-Bcl-xL (ab2764, abcam); rabbit anti-STING(19,851-1-AP, Proteintech); rabbit anti-K63-linkage polyubiquitin (5621, Cell Signaling Technology); rabbit anti-K48-linkage polyubiquitin (8081, Cell Signaling Technology); mouse anti-peroxisome proliferator-activated receptor-γ coactivator-1α (PGC-1α) (66369-1-Ig, Proteintech); rabbit anti- nuclear factor erythroid 2-related factor 2 (nrf2) (ab137550, abcam); rabbit anti-phospho-AMPKα(Thr172) (2535, Cell Signaling Technology); rabbit anti-AMPKα (207442, abcam); rabbit anti-phospho-TANK-binding kinase 1 (TBK1; Ser172) (5483, Cell Signaling Technology); rabbit anti-TBK1 (3504, Cell Signaling Technology); and rabbit anti-β-actin (AF7018, Affinity Biosciences). After being rinsed in TBST three times, the membrane was incubated with the corresponding horseradish-peroxidase conjugated secondary antibody (Millipore) for 1 h at room temperature. The blots were visualized using an enhanced chemiluminescence fluid (Millipore).

### Animal treatment, RPE flat mount and immunohistochemistry

Male BALB/c albino mice aged 8 to 10 weeks were kept on a 12 h dark/light cycle, with plenty food and water. The shRNA-SHP-1-rLV specific to murine SHP-1 (Taitool Bioscience) was used to knockdown SHP-1 in murine RPE (mRPE) in vivo. The target sequence of murine SHP-1 was 5’-GGACATTTCTTGTGCGTGA-3’.We gave a subretinal injection of 1μL of shRNA-SHP-1-rLV into the left eyes. The right eyes received an equivalent volume of scramble-rLV as control. After 2 weeks, the mice were sacrificed and eyes were enucleated and fixed in 4% formaldehyde for 30 min. The RPE flat mounts were prepared as described previously with some modification (Felszeghy et al. 2019). The mounts were treated with 5% goat serum and 0.3% Triton X-100 for 1 h, and then incubated with primary antibodies at 4 °C overnight: mouse anti-SHP-1(610125, BD Biosciences); rabbit anti-TOMM20 (ab186725, abcam) and rabbit anti-ZO-1 (61-7300, Invitrogen). After brief rinses, the mounts were incubated with corresponding secondary antibodies (Invitrogen) for 1 h at room temperature. The mounts were washed and used for confocal microscopy investigation.

### Statistical analysis

Data are presented as the mean ± standard deviations of at least three independent experiments. Statistical analyses were performed with SPSS (version 17; IBM, Armonk, NY, USA). Statistical significance was evaluated by Student’s *t* test, one-way ANOVA or two-way ANOVA, followed by Dunnett's multiple comparisons test. Differences were considered statistically significant at *P* < 0.05.

## Results

### SHP-1 knockdown aggravates cell death and mitochondrial dysfunction in RPE cells exposed to atRAL

**Fig. 1:**
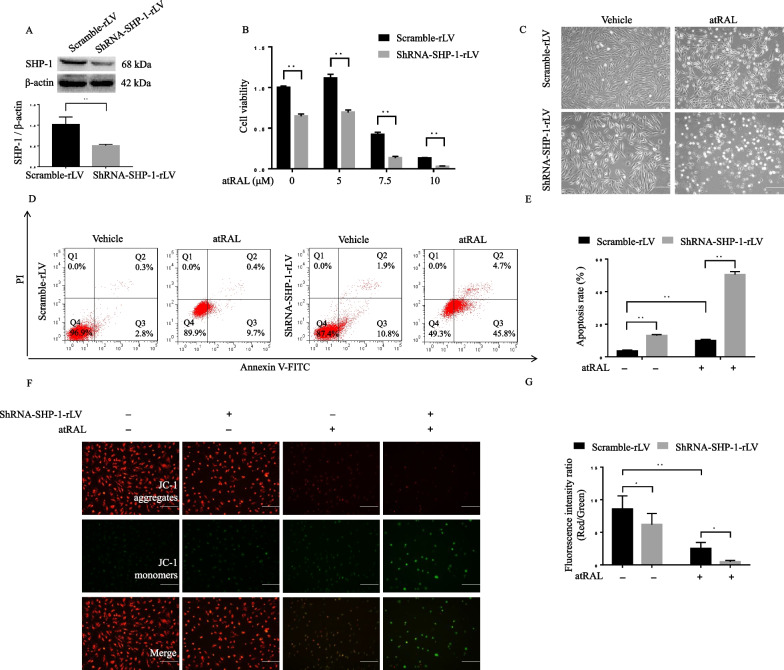
SHP-1 knockdown exacerbates atRAL-induced mitochondria-dependent apoptosis. **A** The knockdown efficiency of shRNA-SHP-1-rLV was confirmed by western blotting. **B** The scramble-rLV- and shRNA-SHP-1-rLV-transfected ARPE-19 cells were exposed to atRAL (0, 5, 7.5, 10 μM, 24 h), and cell viability was measured using a CCK-8 assay. **C** Light microscopic images of cell morphology after exposure to atRAL (7.5 μM, 24 h). Scale bar = 100 μm. **D**, **E** Representative flow cytometry images of Annexin V-FITC/PI staining (**D**) and quantitative analysis of apoptosis rates (Annexin V-FITC + /PI- and Annexin V-FITC + /PI + cells) (**E**). **F**, **G** Cells were stained with JC-1 dyes, after being exposed to atRAL (7.5 μM, 12 h) (**F**), and the red/green fluorescence ratios were calculated (**G**). Scale bar = 100 μm. *P < 0.05 and **P < 0.01

We first transfected ARPE-19 cells with shRNA-SHP-1-rLV to elucidate the effects of SHP-1 knockdown on the viability of RPE cells. The knockdown efficiency was determined by western blotting analysis (Fig. 1A). As shown in Fig. 1B, the viability of shRNA-SHP-1-rLV-transfected cells, was about 40% lower than that of scramble-rLV transfected cells. Exposure to atRAL decreased the viability of ARPE-19 cells at concentrations of 7.5 or 10 μM; this effect was aggravated by SHP-1 knockdown**.** Morphologically, atRAL-induced damage was evident as cytoplasm shrinkage. SHP-1 knockdown significantly worsened the morphological changes (Fig. 1C). Annexin V-FITC/PI staining revealed that treatment with atRAL (7.5 μM) for 12 h resulted in an apoptosis rate of 10% (both Annexin V + /PI- and Annexin V + /PI + cells) of ARPE-19 cells. SHP-1 knockdown significantly increased the apoptosis rate to approximately 50% in cells exposed to atRAL (Fig. 1D, E). Next, we examined the MMP by performing JC-1 staining. The control ARPE-19 cells showed intense red fluorescence, which indicated the maintenance of MMP polarization. However, atRAL significantly attenuated the red fluorescence and enhanced the green fluorescence, indicating collapse of the MMP. SHP-1 knockdown elicited a slight decrease in the ratio of red/green fluorescence before exposure to atRAL, and a significantly greater collapse in MMP after cells were exposed to atRAL (Fig. 1F, G).

**Fig. 2:**
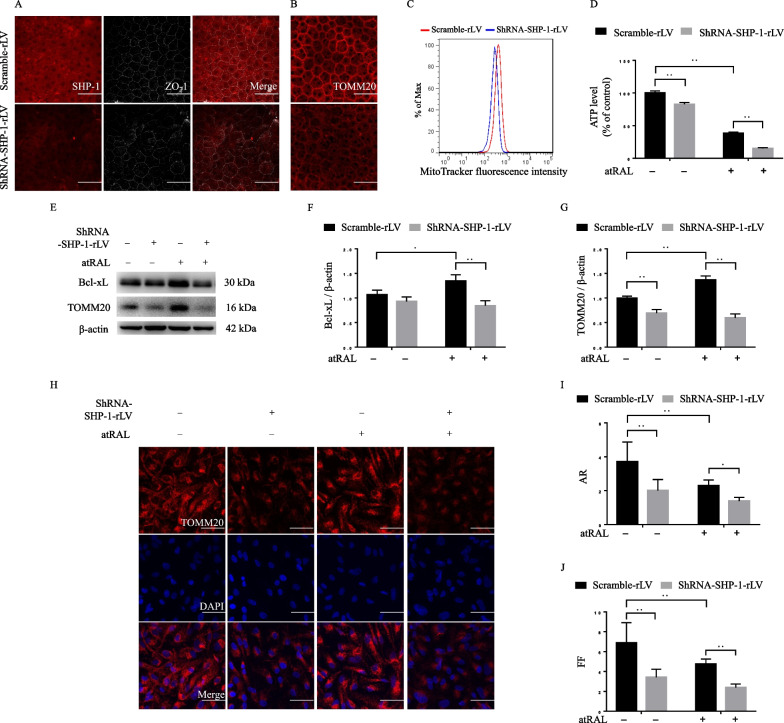
SHP-1 knockdown induces mitochondrial dysfunction. **A**,**B** Immunofluorescent images of scramble-rLV- and shRNA-SHP-1-rLV-transfected murine RPE flat mounts, stained for SHP-1/ZO-1 (**A**), and TOMM20 (**B**). Scale bar = 50 μm. **C** Abundance of mitochondria determined by MitoTracker fluorescence intensities in scramble-rLV- and shRNA-SHP-1-rLV-transfected ARPE-19 cells. The scramble-rLV- and shRNA-SHP-1-rLV-transfected ARPE-19 cells were treated with atRAL (7.5 μM, 24 h). **D** Relative ATP levels. **E–G** Western blotting analysis of the relative expression levels of Bcl-xL (**F**) and TOMM20 (**G**). Representative immunofluorescent images of ARPE-19 cells labeled with TOMM20 were shown in (**H**). Scale bar = 50 μm. The AR values (**I**) and FF values (**J**) were calculated and compared among different groups. *P < 0.05 and **P < 0.01

We then performed the immunofluorescent staining of mRPE flat mount. In the control group, the regular hexagonal shape of the mRPE monolayer was delineated by the staining of the tight junction protein ZO-1, and SHP-1 was mainly located in the cytoplasm of the mRPE cells in vivo. Interestingly, the shRNA-SHP-1-rLV-transfected mRPE cells were irregular in shape. The staining of ZO-1 was relatively disorganized and discontinuous somewhere, suggesting the disrupted barrier function (Fig. 2A). Given the vital role of mitochondria in RPE, we then investigated whether SHP-1 affected mitochondria in mRPE cells in vivo. As shown in Fig. 2B, the expression of the mitochondrial marker TOMM20 was downregulated by SHP-1 knockdown in mRPE. Consistently, knockdown of SHP-1 reduced the mitochondrial mass in ARPE-19 cells, as determined by mitochondrial labeling with MitoTracker dyes (Fig. 2C). Knockdown of SHP-1 dampened basal ATP production. atRAL-induced energy metabolism dysfunction was further exacerbated by SHP-1 knockdown (Fig. 2D). Intriguingly, the expression levels of Bcl-xL and TOMM20 were slightly upregulated in cells exposed to atRAL, which might constitute an adaptive response for self-protection from further damage. Knockdown of SHP-1 significantly suppressed the expression levels of these proteins (Fig. 2E-G). In addition, we used the immunofluorescent staining of TOMM20 to study the changes in mitochondrial dynamics. The mitochondria morphology was reflected by AR/FF values, which are indicators of dynamics of mitochondrial network. As depicted in Fig. 2H, the tubular and filamentous mitochondria in ARPE-19 cells formed a relatively complex and branched network at basal conditions. However, atRAL treatment induced the shortening of partial region of mitochondria. SHP-1 knockdown significantly decreased the immunofluorescence of TOMM20, which was in accordance with the finding in Fig. 2E. Moreover, SHP-1 knockdown induced an imbalance in mitochondrial dynamics towards mitochondrial fragmentation before stimulation. atRAL stimulation in the context of SHP-1 knockdown exacerbated the fragmentation, evidenced by the punctiform shape of most mitochondria. The analysis of AR/FF values further supported our observations (Fig. 2I, J).These results imply that SHP-1 knockdown accelerates atRAL-induced mitochondria-dependent apoptosis, and aggravates mitochondrial dysfunction.

### SHP-1 knockdown augments the posttranslational modification and activation of STING

**Fig. 3:**
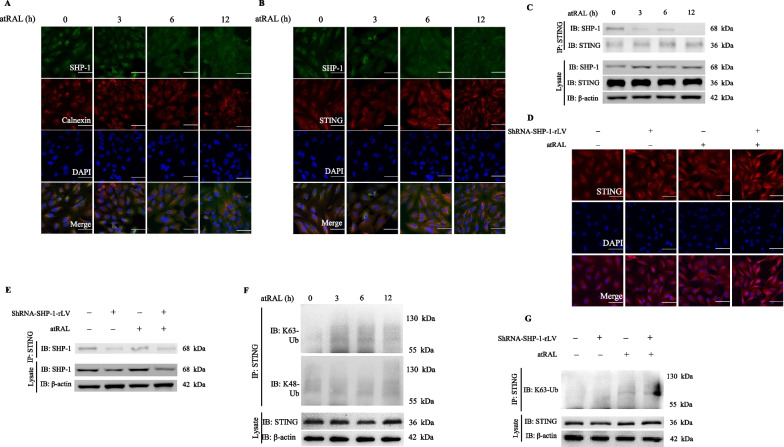
SHP-1 interacts with STING and suppresses its activation through posttranslational modifications. **A**–**C** ARPE-19 cells were exposed to atRAL (7.5 μM, 0, 3, 6, 12 h). Immunofluorescent images of cells costained for SHP-1 and calnexin (**A**) or for SHP-1 and STING (**B**) were shown. Scale bar = 50 μm. **C** Immunoprecipitation and western blotting of cell lysates. **D**, **E** Scramble-rLV- and shRNA-SHP-1-rLV-transfected ARPE-19 cells were treated with atRAL (7.5 μM, 6 h), and subject to immunofluorescent staining for STING (**D**) or immunoprecipitation analysis (**E**). Scale bar = 50 μm. **F** ARPE-19 cells were incubated with atRAL (7.5 μM, 0–12 h), and the ubiquitination of STING was detected by immunoprecipitation. **G** the K63- ubiquitination of STING in the scramble-rLV- and shRNA-SHP-1-rLV-transfected ARPE-19 cells exposed to atRAL (7.5 μM, 6 h)

To further investigate the underlying regulatory mechanism of SHP-1 in RPE cells, we studied the subcellular localization of SHP-1. The immunofluorescent staining of SHP-1 in basal conditions revealed its cytoplasmic distribution with more prominent perinuclear aggregation. It was partially colocalized with calnexin, an ER marker. However, the perinuclear aggregation of SHP-1 was dramatically decreased in cells upon atRAL treatment (Fig. 3A). Because STING, a critical adaptor protein residing in the ER, was reported to be aberrantly activated in the degenerating RPE of eyes with AMD (Kerur et al. 2018), we examined whether SHP-1 interacted with STING in our cellular model. Immunofluorescence of STING revealed its retention in the ER and an overlap with SHP-1 localization before stimulation. Nevertheless, STING formed aggregated specks in the perinuclear region, reaching a peak at 6 h after exposure to atRAL, a hallmark of STING activation (Ishikawa et al. 2009). Interestingly, the colocalization of SHP-1 and STING was weakened by exposure to atRAL (Fig. 3B). The endogenous immunoprecipitation analysis confirmed an interaction between SHP-1 and STING in ARPE-19 cells that was significantly repressed by atRAL (Fig. 3C). This implies that SHP-1 likely regulates the activation of STING in RPE cells.

Next, we examined the activation of STING in shRNA-SHP-1-rLV-transfected cells. Surprisingly, we observed discrete spontaneous perinuclear aggregations of STING even before exposure to atRAL, and administration of atRAL elicited more pronounced accumulation (Fig. 3D). The immunoprecipitation analysis showed that the interaction between SHP-1 and STING was blocked in shRNA-SHP-1-rLV-transfected cells (Fig. 3E). Previous studies have shown that a series of posttranslational modifications regulate the activity of STING; for example, K63-linked ubiquitination facilitates the aggregation of STING and subsequent signal transduction (Ye et al. 2019a), and K48-linked ubiquitination accelerates the degradation of STING (Zhong et al. 2009). In our study, we found that exposure to atRAL dramatically increased the K63-linked ubiquitination of STING. By contrast, K48-linked ubiquitination was induced very mildly (Fig. 3F). Moreover, SHP-1 knockdown markedly increased atRAL-induced K63-linked ubiquitination of STING at 6 h after exposure to atRAL (Fig. 3G). Taken together, our results imply that SHP-1 knockdown facilitates K63-linked ubiquitination of STING and induces its overactivation.

### STING inhibition mitigates atRAL-induced mitochondria-dependent apoptosis

**Fig. 4:**
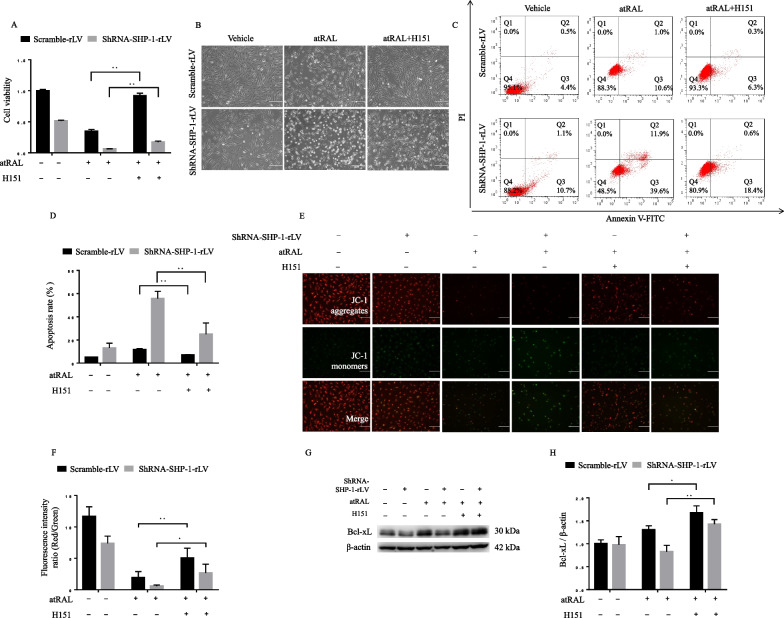
Effects of SHP-1 knockdown on cell apoptosis are attenuated by the inhibition of STING. Scramble-rLV- and shRNA-SHP-1-rLV-transfected ARPE-19 cells were treated with atRAL (7.5 μM), with or without pretreatment with the STING antagonist, H151 (1 μM), for the indicated time. **A** Viability of ARPE-19 cells (atRAL, 24 h) was examined using a CCK-8 assay. **B** Light microscopic images of the morphological features of ARPE-19 cells (atRAL, 24 h). Scale bar = 100 μm. **C**, **D** Representative images of ARPE-19 cells (atRAL, 12 h) stained with Annexin V-FITC/ PI (**C**), and quantitative analysis of the apoptosis rates (**D**). **E**, **F** ARPE-19 cells (atRAL, 12 h) were stained with JC-1 dyes (**E**), and the fluorescence intensity ratios (red/green) were calculated (**F**). Scale bar = 100 μm. **G**, **H** Western blotting analysis of Bcl-xL expression levels in ARPE-19 cells (atRAL, 24 h). *P < 0.05 and **P < 0.01

Next, we investigated whether STING is involved in atRAL-induced apoptosis of ARPE-19 cells in the context of SHP-1 knockdown. H151, a covalent antagonist targeting human STING, inhibits the palmitoylation of STING at Golgi bodies and subsequent signaling transduction (Haag et al. 2018). As shown in Fig. 4A, B, inhibiting STING with H151 significantly improved the viability of control and SHP-1 knockdown cells exposed to atRAL, as determined by CCK-8 assays and cell morphology under a light microscope. Results of flow cytometry indicated that apoptosis induced by atRAL incubation was suppressed markedly by STING inhibition, in both control and SHP-1 knockdown cells (Fig. 4C, D) Accordingly, the MMP of cells exposed to atRAL was better preserved by the inhibition of STING, with an increased red/green fluorescence ratio (Fig. 4E, F). Moreover, the suppression of STING significantly upregulated the Bcl-xL expression (Fig. 4G, H). These results suggest that the activation of STING, augmented by SHP-1 knockdown, plays an essential role in atRAL-induced mitochondria- dependent apoptosis of ARPE-19 cells.

### Inhibition of STING promotes SHP-1 knockdown-suppressed mitochondrial biogenesis

**Fig. 5:**
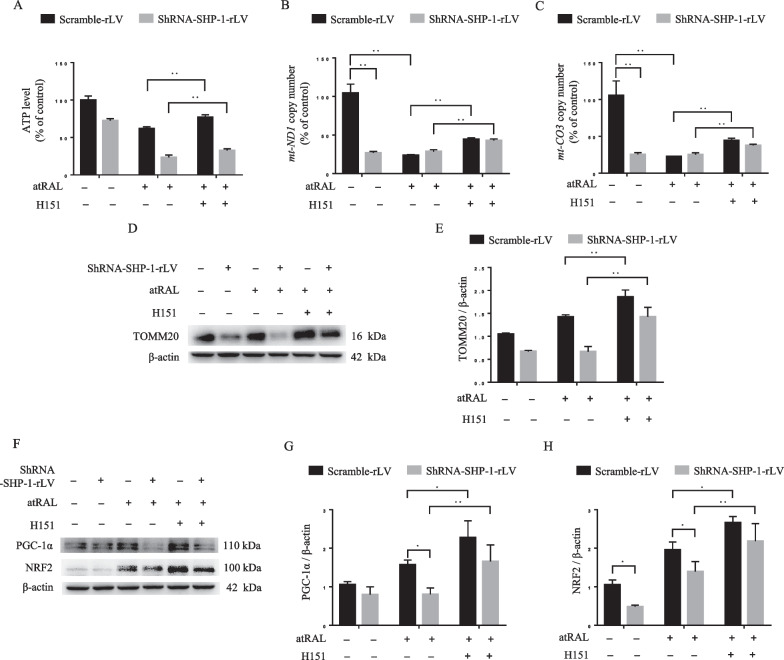
Inhibition of STING reverses mitochondrial biogenesis suppressed by SHP-1 knockdown. Scramble-rLV- and shRNA-SHP-1-rLV- transfected ARPE-19 cells were incubated with atRAL (7.5 μM, 24 h), with or without H151 (1 μM). **A** The relative ATP levels in different groups were measured. mtDNA content, represented by *mt -ND1* (**B**), and *mt-CO3* (**C**) was assessed by qPCR. **D**–**H** Western blotting analysis of TOMM20 (**D**, **E**), PGC-1α (**F**,** G**), and nrf2 (**F**,** H**). *P < 0.05 and **P < 0.01

Mitochondrial biogenesis is the process of mitochondrial renewal that is dynamically regulated by the coordination of nDNA/mtDNA, and a series of transcriptional factors. Disruption of mitochondrial biogenesis could lead to mitochondrial dysfunction. As shown in Fig. 5A, the inhibitory effects of atRAL and/or SHP-1 knockdown on ATP production were reversed by the inhibition of STING. Furthermore, SHP-1 knockdown and exposure to atRAL significantly downregulated the copy numbers of *mt-ND1* and *mt-CO3*, suggesting the loss of mitochondrial genomes (Fig. 5B, C). However, H151 significantly increased the copy numbers of *mt-ND1* and *mt-CO3*. Inhibition of STING markedly increased the expression of TOMM20 in control and shRNA-SHP-1-rLV-transfected cells (Fig. 5D, E). We then determined the expression levels of the critical transcriptional factors involved in mitochondrial biogenesis (PGC-1α and nrf2). The results revealed that the expression levels of PGC-1α and nrf2 were upregulated by atRAL. This may represent an adaptive response compensating for the destruction of mitochondrial genomes. Nevertheless, this response was weaker in shRNA-SHP-1-rLV-transfected cells compared with the control cells. Inhibition of STING significantly enhanced the expression of PGC-1α and nrf2 in control and shRNA-SHP-1-rLV-transfected cells (Fig. 5F–H). Taken together, these findings suggest that SHP-1 knockdown inhibits the process of mitochondrial biogenesis, at least in part, by facilitating the activation of STING.

### The AMPK pathway is involved in STING-regulated mitochondrial biogenesis and cell survival

**Fig. 6:**
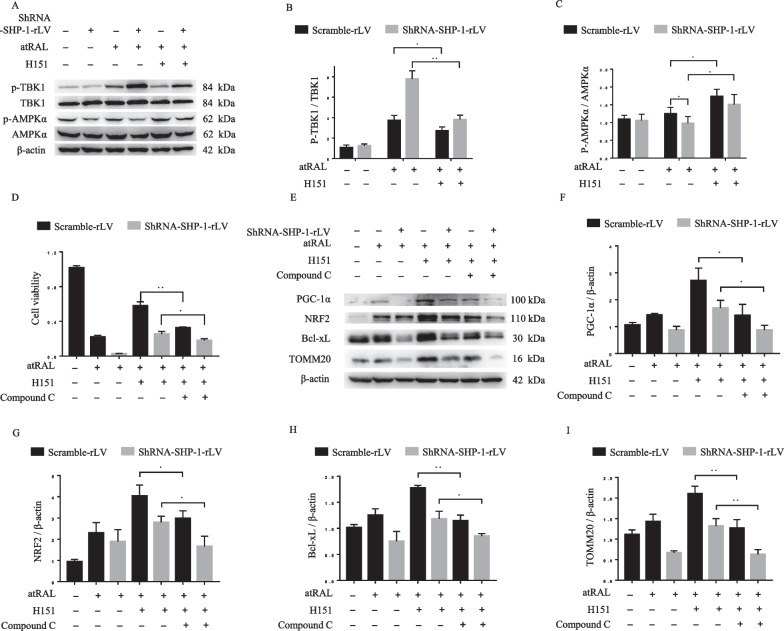
Inhibition of AMPK partially abrogates the effects of STING inhibition. **A**–**C** Western blotting of phosphorylated TBK1/TBK1 (**B**) and phosphorylated AMPKα/AMPKα (**C**) in ARPE-19 cells exposed to atRAL (7.5 μM, 24 h), with or without H151(1 μM). **D** Viability of scramble-rLV- and shRNA-SHP-1-rLV-transfected ARPE-19 cells exposed to atRAL (7.5 μM, 24 h), after pretreatment with Compound C (5 μM) and H151 (1 μM) as indicated. **E-I** Western blotting analysis of PGC-1α (**F**), nrf2 (**G**), Bcl-xL (**H**) and TOMM20 (**I**). *P < 0.05 and **P < 0.01

Previous studies indicate that TBK1 lies downstream of STING in the signal transduction pathway (Tanaka and Chen 2012). AMPK mediates the adaptive responses to oxidative stress (Wu et al. 2014), regulates various aspects of mitochondrial homeostasis, including mitochondrial biogenesis (Herzig and Shaw 2018), and acts as the downstream target of the STING/TBK1 complex (Zhao et al. 2018). Therefore, we examined whether the AMPK pathway participates in the regulatory effects of STING on mitochondrial biogenesis and cell survival. As shown in Fig. 6A-C, western blotting revealed that atRAL-induced phosphorylation of TBK1 was significantly enhanced by SHP-1 knockdown, and this effect was attenuated by H151 (Fig. 6A, B). The phosphorylation of AMPKα (Thr172), which is necessary and sufficient for AMPK activation (Stein et al. 2000), was significantly increased by the inhibition of STING in control and shRNA-SHP-1-rLV-transfected cells (Fig. 6A, C). To demonstrate the roles of the AMPK pathway in STING-regulated cell survival and mitochondria, we treated cells with Compound C, a specific AMPK antagonist. As presented in Fig. 6D, the protective effects of H151 on cell viability after exposure to atRAL were significantly attenuated by Compound C. Moreover, the expression levels of PGC-1α, nrf2, Bcl-xL, and TOMM20, which are markers of mitochondrial biogenesis and cell survival, were enhanced by STING inhibition, but the effects of STING inhibition were attenuated by AMPK inhibition (Fig. 6E-I). Overall, these findings indicate that the beneficial effects of STING inhibition are at least partly reliant on increased AMPK activity.

## Discussion

**Fig. 7:**
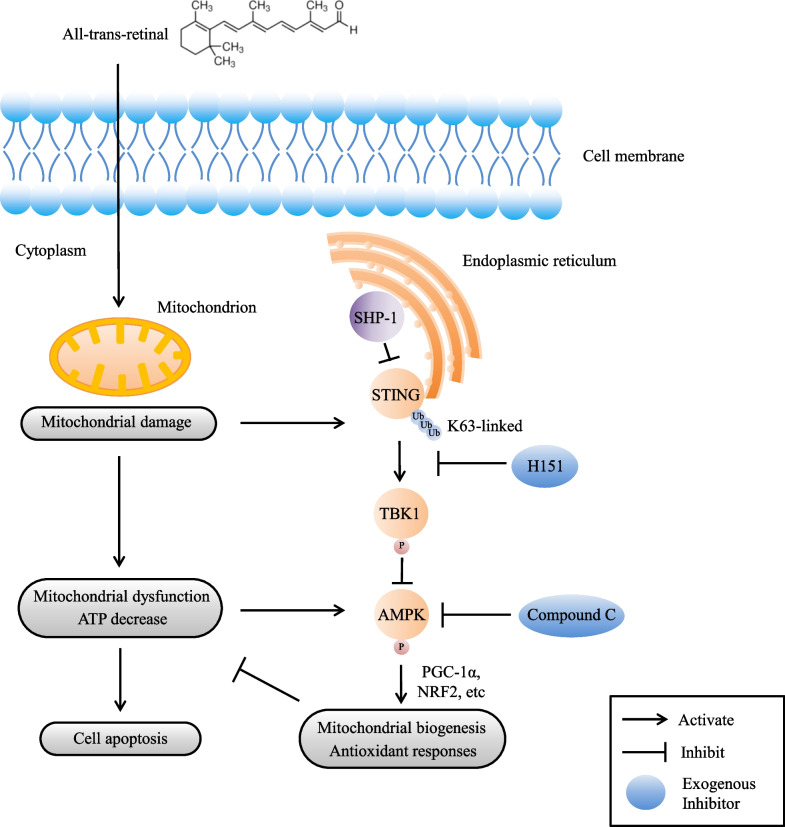
Schematic diagram of the proposed model  for atRAL-elicited responses within RPE cells regulated by SHP-1. atRAL causes mitochondrial damage, contributes to the production of ROS and leads to energy insufficiency in RPE cells. The energy stress-induced AMPK activation elicits adaptive protective responses to counterbalance mitochondrial dysfunction. However, the damaged mitochondria trigger K63-linked ubiquitination, aggregation and activation of STING in the ER, which phosphorylates TBK1, and subsequently suppresses AMPK activity. SHP-1 interacts with STING and restrains its activity. SHP-1 loss results in the overactivation of STING, dampening of mitochondrial biogenesis and antioxidant defense, and finally aggravation of oxidative stress-induced death in RPE cells

The progression of AMD involves predominantly mitochondrial damage in the RPE layer rather than the neural retina, which suggests that mitochondria in the RPE are a vital target for protective therapies (Terluk et al. 2015). SHP-1 is a critical regulator of multiple cellular signaling cascades, mainly by dephosphorylation, and it is also demonstrated to regulate reactive oxygen species (ROS) production (Krotz et al. 2005; Gruber et al. 2015). Mitochondria, the primary source of cellular ROS, are intimately linked to cellular energy and cell fate. However, the impact of SHP-1 on mitochondrial health has not been fully elucidated. Therefore, in the present study, we examined the effects of SHP-1 knockdown on the mitochondria in RPE cells following atRAL-induced oxidative stress. The results showed that SHP-1 knockdown markedly accelerated mitochondria-dependent apoptosis, aggravated mitochondrial dysfunction induced by atRAL, and disrupted mitochondrial biogenesis. Moreover, atRAL activated STING, which was enhanced by SHP-1 knockdown, whereas inhibition of STING significantly alleviated cellular injury and augmented mitochondrial biogenesis, which was at least in part mediated by the AMPK signaling pathway (Fig. 7). Overall, our results indicated that SHP-1 regulates mitochondrial health in RPE cells possibly through the STING/TBK1/AMPK signaling pathways.

In the first part of our study, we demonstrated that atRAL, a potent oxidative stressor, induced apoptosis and MMP collapse in RPE cells, which were further exacerbated by SHP-1 knockdown. Previous studies have shown that the mitochondria in aged RPE cells exhibit structural and functional abnormalities, which contribute to their vulnerability to oxidative stress and the increased risk of AMD (Feher et al. 2006; He and Tombran-Tink 2010). The decreased ATP level, for example, weakens the capacity of RPE cells to cope with pathological stimuli (Schutt et al. 2012). In our model, SHP-1 knockdown resulted in decreases in MMP, ATP production, abundance of mitochondria, and expression of the mitochondrial marker TOMM20. These changes are analogous to the phenotype of aged RPE cells. Bcl-xL, a member of the pro-survival Bcl-2 family, is known for its role in preventing MMP loss and the death of RPE cells (Zhang et al. 2007). Consistently, we found that SHP-1 knockdown resulted in downregulation of Bcl-xL and contributed to mitochondria-dependent apoptosis in response to oxidative stress. Hence, SHP-1 loss drives mitochondrial dysfunction, which may underlie the susceptibility of RPE cells to oxidative stress-induced damage and death.

The abundance of mitochondria is maintained by a balance between biosynthesis and elimination (i.e., mitophagy). Because we did not find evidences for increased autophagy after SHP-1 knockdown (data not shown), we investigated its effects on mitochondrial biogenesis. The upregulation of transcriptional factors, especially PGC-1α and NRFs, is pivotal to  coordinated expression of the nuclear and mitochondrial genes involved in mitochondrial biogenesis (Wu et al. 1999). Mitochondrial biogenesis was shown to protect against oxidative damage in RPE cells (Hua et al. 2018; Sreekumar et al. 2016). Meanwhile, nrf2 activates antioxidant defense pathways to aid cell survival (Wu et al. 2021). It has been reported that PGC-1α/nrf2 double-knockout mice exhibit severe RPE degeneration (Felszeghy et al. 2019). In our study, knockdown of SHP-1 significantly inhibited the expression of PGC-1α/nrf2 and impaired mitochondrial biogenesis in RPE cells. Furthermore, TOMM20, a key component of the mitochondrial transport machinery, is controlled by mitochondrial biogenesis via NRFs, and has reciprocal effects on mitochondrial biogenesis by delivering nuclear-encoded proteins to the mitochondria (Blesa et al. 2007). TOMM20 is also essential for ATP production, MMP maintenance (Park et al. 2019), and defense against oxidative stress (Zhang et al. 2010). We found that TOMM20 was downregulated by SHP-1 knockdown, consistent with a previous study of degenerating RPE cells in vivo (Brown et al. 2019). These findings indicate that the mitochondrial dysfunction caused by SHP-1 knockdown is possibly due to defects in mitochondrial biogenesis and antioxidant defense.

Next, we sought to clarify the mechanisms by which SHP-1 knockdown suppresses mitochondrial biogenesis. We found that, in ARPE-19 cells, SHP-1 is localized mainly in the ER, similar to others’ finding in A431 cells (Tenev et al. 2000). Because SHP-1 mainly exerts its effects by dephosphorylating the interacting proteins nearby, we assumed that the modified activity of an ER-resident effector may account for the effects of SHP-1 knockdown. We confirmed an interaction between SHP-1 and ER-resident STING, and that the knockdown of SHP-1 increased K63-linked ubiquitination of STING, which could facilitate the aggregation and translocation of STING to elicit full signal transduction (Ye et al. 2019b). This is consistent with the most recent finding that SHP-1, as a member of the protein tyrosine phosphatase family, suppressed the K63-linked ubiquitination of STING by directly dephosphorylating STING at Tyr162 in HEK293T cells (Wang et al. 2022). STING is activated by binding of released mtDNA to cyclic GMP-AMP synthase (Fang et al. 2016). However, there is little evidence to indicate whether and how STING regulates mitochondrial homeostasis in a reciprocal manner. Until recently, the inhibition STING is demonstrated to enhance the mtDNA content and ameliorate cisplatin-induced renal mitochondrial dysfunction (Gong et al. 2021). We have revealed that the inhibition of STING promoted the survival of RPE cells. Even in the context of SHP-1 knockdown, the inhibition of STING upregulated mitochondrial biogenesis to mitigate the mitochondrial dysfunction. Overall, these findings indicate that, the overactivation of STING, promoted by SHP-1 knockdown, suppresses mitochondria biogenesis in RPE cells.

Previous studies have shown that AMPK activation plays a central role in mitochondrial biogenesis and mitochondrial ROS reduction, both of which are mediated by PGC-1α (Rabinovitch et al. 2017; Herzig and Shaw 2018). Additionally, AMPK upregulates the expression of the antiapoptotic protein Bcl-xL (Cao et al. 2010). Recently, it was demonstrated that the AMPK pathway is a downstream target of STING/TBK1 (Peng et al. 2020). Therefore, in the final part of our study, we examined whether the AMPK pathway is involved in STING-regulated mitochondrial biogenesis and cell survival in RPE cells. We demonstrated that the phosphorylation of AMPK in our model was further enhanced by H151. The effects of STING on cell survival and mitochondrial biogenesis were attenuated by the inhibition of AMPK. Thus we conclude that the AMPK pathway, which is negatively regulated by STING activation, contributes to cell survival and mitochondrial biogenesis in RPE cells. Because AMPK regulates other cellular events including autophagy initiation/progression, inflammation suppression, for example (Herzig and Shaw 2018; Peng et al. 2020), it will be necessary to investigate whether these cellular processes are involved in the protective effects of AMPK activation in RPE cells.

## Conclusions

In summary, our study has revealed SHP-1 interacts with STING and inhibits its K63-linked ubiquitination in RPE cells. Overactivation of STING, as induced by SHP-1 knockdown, disrupts mitochondrial biogenesis, and accelerates mitochondrial decay and mitochondrial dysfunction by inhibiting the AMPK pathway. These events increase the susceptibility of RPE cells to oxidative stress. Overall, these findings imply that the SHP-1-STING-AMPK axis might be a promising therapeutic target in degenerative retinal diseases.

## Data Availability

The datasets used and/or analysed during the current study are available from the corresponding author on reasonable request.

## Abbreviations

RPE: Retinal pigment epithelium
AMD: Age-related macular degeneration
atRAL: All trans retinal
SHP-1: Src-homology 2 domain-containing phosphatase-1
STING: Stimulator of interferon genes
AMPK: Adenosine monophosphate-activated protein kinase
ER: Endoplasmic reticulum
shRNA: Short hairpin RNA
TOMM20: Translocase of outer mitochondrial membrane 20
*mt-ND1*: Mitochondria-encoded NADH dehydrogenase subunit 1
*mt-CO3*: Mitochondria-encoded cytochrome c oxidase III
PGC-1α: Peroxisome proliferator-activated receptor-γ coactivator-1α
nrf2: Nuclear factor erythroid 2-related factor 2
TBK1: TANK-binding kinase 1
ZO-1: Zonula occludens-1
